# Asprellcosides B of *Ilex asprella* Inhibits Influenza A Virus Infection by Blocking the Hemagglutinin- Mediated Membrane Fusion

**DOI:** 10.3389/fmicb.2018.03325

**Published:** 2019-01-23

**Authors:** Wen Zhang, Si-Tai Chen, Qiu-Yan He, Li-Quan Huang, Xiong Li, Xiao-Ping Lai, Shao-Feng Zhan, Hui-Ting Huang, Xiao-Hong Liu, Jianguo Wu, Geng Li

**Affiliations:** ^1^Laboratory Animal Center, Guangzhou University of Chinese Medicine, Guangzhou, China; ^2^The First Affiliated Hospital of Zhejiang Chinese Medical University, Hangzhou, China; ^3^Guangdong Provincial Academy of Chinese Medical Sciences, Guangzhou, China; ^4^Dongguan Mathematical Engineering Academy of Chinese Medicine, Guangzhou University of Traditional Chinese Medicine, Dongguan, China; ^5^The First Affiliated Hospital of Guangzhou University of Chinese Medicine, Guangzhou, China; ^6^Key Laboratory of Virology, Institute of Medical Microbiology, Jinan University, Guangzhou, China; ^7^Guangdong Longfan Biological Science and Technology Company, Ltd., Foshan, China

**Keywords:** triterpenoid saponins sulfate, *Ilex asprella*, anti-influenza activity, hemagglutinin (HA) protein, competitive inhibition, inhibition of membrane fusion

## Abstract

*Ilex asprella* is routinely used in China as a traditional medicinal herb to treat influenza (Flu). However, its specific antiviral activity and underlying molecular mechanism have not yet been determined. In this study, we sought to determine the antiviral activity and mechanism of Asprellcosides B, an active component extracted from *Ilex asprella*, and used against the influenza A virus cell culture. We also performed a computer-assisted structural modeling analysis and carried out surface plasmon resonance (SPR) experiments in the hope of determining the viral target of Asprellcosides B. Results from our studies show that Asprellcosides B reduced virus replication by up to 63% with an IC50 of about 9 μM. It also decreased the low pH-induced and virus-mediated hemolysis by 71% *in vitro*. Molecular docking simulation analysis suggested a possible binding of Asprellcosides B to the hemagglutinin (HA), which was confirmed by a surface plasmon resonance (SPR) assay. Altogether, our findings demonstrate that Asprellcosides B inhibits the influenza A virus, through a specific binding to the HA, resulting in the blockade of the HA-mediated membrane fusion.

## Introduction

The Influenza A virus infects hundreds of millions of people worldwide every year, posing a great threat to global health. Belonging to the *Orthomyxoviridae* family, the Influenza A virus contains a segmented and single-stranded RNA genome of negative polarity (International Committee on Taxonomy of Viruses, 2012). Historically, amantadine (Davies et al., 1964; Hay et al., 1985) and rimantadine (Dolin et al., 1982; Wintermeyer and Nahata, 1995) were used to treat influenza. However, the rapid emergence of drug-resistant mutants have limited their use in clinics (Dong et al., 2015). Currently, neuraminidase (NA) inhibitors such as Zanamivir (Woods et al., 1993) and Oseltamivir (Eisenberg et al., 1997) are mainly used as antiviral drugs for the treatment of influenza. The drug-resistance to NA inhibitors represents a new challenge for antiviral therapies treating influenza (Huang et al., 2018), even though Peramivir (Babu et al., 2010) and Laninamivir (Yamashita et al., 2009) are effective against Zanamivir- and/or Oseltamivir-resistant influenza viruses. Therefore, there is an unmet medical need to discover and develop new classes of antiviral drugs to control influenza (Hayden, 2006; Król et al., 2014). Traditional Chinese medicine may serve as an alternative to identify novel antiviral drugs (Wang et al., 2006; Chattopadhyay et al., 2009; Ge et al., 2010).

*Ilex asprella* is comprised of a variety of Aquifoliaceae, found in different regions across China (Du et al., 2017). It has been routinely used in China as a Chinese herbal medicine to treat the “common cold”. Previous studies found that its main components include triterpenoid saponins, phenolic acids, and alkaloids (Huang et al., 2012; Lei et al., 2014). Several recent studies demonstrated the anti-influenza activity of triterpenoid saponin (Li et al., 2007; Song et al., 2016; Gong et al., 2017). The antiviral activity of *Ilex asprella* extracts was also demonstrated in an animal model of influenza A virus infection (Peng et al., 2016).

In this study, we have extracted pure Asprellcosides B from *Ilex asprella*, using phytochemistry and chromatographic methodologies (Porath and Flodin, 1959; Lerman, 1987) and evaluated its anti-influenza virus activity. We have also determined the HA as the viral target of Asprellcosides B, based on findings from a molecular docking simulation (Wang et al., 2015, 2016; Makau et al., 2017) and surface plasmon resonance (SPR) analysis (Giannetti, 2011). These results suggest that Asprellcosides B blocks the HA-mediated cell entry of the influenza A virus.

## Materials and Methods

### Plant Material

The radix and stems of a selection of *Ilex asprella* (Hook. Et Arn.) Champ. Ex Benth (Eisenberg et al., 1997) was taken from a commercial plantation situated in Longyan city in Fujian Province, China. The plant material was dried without delay in a vacuum decompression drying oven at 60°C for 3 h, to a moisture content of less than 13% and then pulverized by a muller (YoN GLI).

### Preparation of Asprellcosides B

Plant material (20 kg) was extracted four times with 70% Ethyl alcohol (EtOH) (4 × 40 L/12 h, 25°C) under reflux, and evaporated under reduced pressure to obtain a residue (638g). The residue was resuspended with water (4 L) and extracted three times with Ethyl acetate (EtOAc) (3 × 638 mL, 25°C), and incubated each time at room temperature for 1 h. The EtOAc-soluble fraction (185 g) was subjected to AB-8 macroporous resin with distilled water, until the eluent was colorless. It was then washed with a gradient elution with EtOH (0.74 L) (20, 50, 70, and 95%) to afford four fractions. Fraction 2 (elution with 50% EtOH) was evaporated under reduced pressure to obtain a residue (86 g). The residue was dissolved in (Methanol, MeOH) and subjected to column chromatography on a Sephadex LH-20 column (MeOH, 100%, 0.43 L) with isocratic elution. The elution was then identified by TLC (Waksmundzka-Hajnos et al., 2008) and the eluent was visualized with a 10% ethanol sulfate solution. Fraction 3, which was visible purplish red by the results of TLC, was collected and enriched, and the product was dried.

**FIGURE 1:**
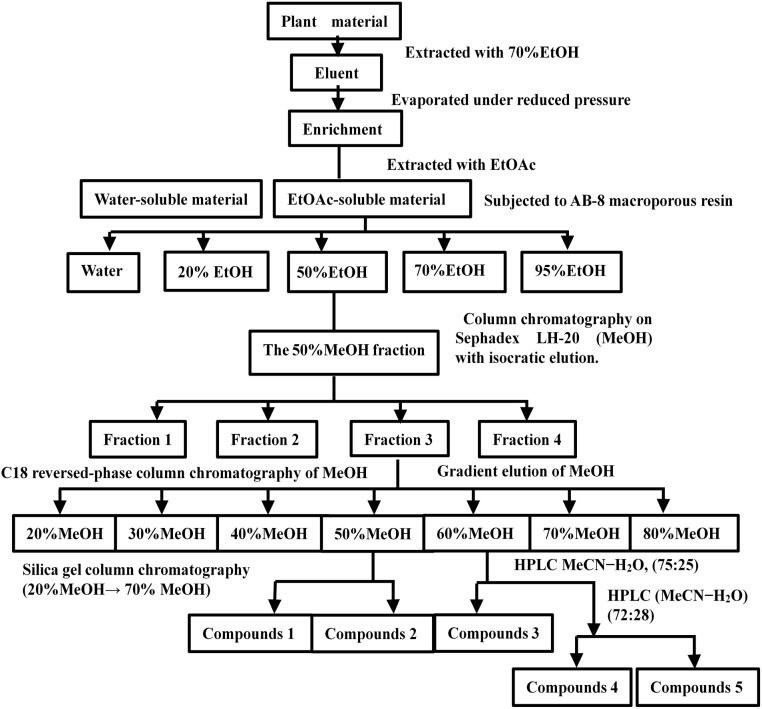
Flow chart of the extraction and isolation of Asprellcosides B.

The product was fractionated by C18 reversed-phase column chromatography (Shinoda et al., 2002) with MeOH (20, 30, 40, 50, 60, 70, and 80%). The fractions were then concentrated and identified by TLC with a 10% ethanol sulfate solution. The fractions that were visible purplish red by TLC were collected and enriched. The fractions (MeOH 50%) were then purified by silica gel column chromatography (20% MeOH to 70% MeOH) to obtain subfractions 1 and 2. The 60% MeOH fraction was purified by (High-performance liquid chromatography, HPLC) (Huang et al., 2018) (MeOH-H_2_O, 75:25) to yield subfractions 3, 4, and 5; subfraction 2 was purified by recrystallization to obtain compound 2 (19.3 mg). Compound 2 was named Asprellcosides B. The flow diagram of extraction and isolation is shown in Figure 1.

### Virus and Cell

A/FM/1/47(H1N1), A/PuertoRico/8/34(H1N1), A/Chicken/Guangdong/1996(H9N2) and A/HongKong/498/97(H3N2) were propagated in an embryonated hen egg. The Zika virus (GenBank accession number, KU955589.1), DENV-2 (TSV01, GenBank accession AY037116) and HSV-1 (Shuwen Wu, Wuhan University, Wuhan, China) were propagated in Vero cells. The viruses were aliquoted and stored at -80°C. Cells (China Center for type culture collection, China) were grown in Dulbecco’s modified Eagle’s medium (DMEM), supplemented with 10% fetal bovine serum, 100 U/mL penicillin, and 100 U/mL streptomycin. Infection medium applied in anti-influenza assays were DMEM, containing 2 μg/mL tosyl phenylalanylchloromethyl ketone (TPCK)-treated trypsin and 1% bovine serum albumin, BSA.

### Anti-influenza Virus Activities of Asprellcosides B

#### Virus Infection

The influenza virus A/FM/1/47 (H1N1) was used in the antiviral assays. A549 Cells were seeded at a density of 2 ×10^4^ cells per well, in the 96-well plates and were maintained at 37°C, 5% v/v CO_2_ in a humidified incubator. Dissolved Asprellcosides B in Dimethylsulfoxide (DMSO) was diluted with serum-free Dulbecco’s Modified Eagle Medium (DMEM), and the final concentration of DMSO was 1%. The viral group and control group received different treatments. The viral group was infected with the influenza virus (50TCID_50_) on confluent monolayers in 100 μL of the infection medium, while the control group was infected on confluent monolayers with 100 μL of the infection medium. Both groups were then incubated at 37°C for 1 h.

#### The Methods of Adding the Drugs

After the virus infection, the media was removed and cells were washed twice with 100 μL of a phosphate buffer saline (PBS) solution at pH7.4. Following that, a twofold gradient of Asprellcosides B and a positive control medicine (Oseltamivir phosphate) were diluted by the infection medium containing 1% DMSO, at an initial concentration of 200 μM in a total of nine gradients. The first row of a 96-well plate was a blank control with normal cells, in 100 μL of the infection medium containing 1% DMSO, while the last row of the plate was a negative control of the cells infected by the virus in 100 μL of the infection medium containing 1% DMSO.

**FIGURE 2:**
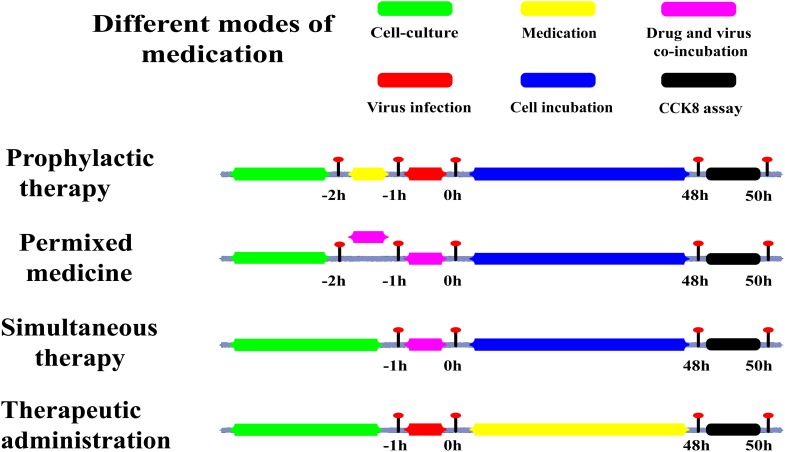
Sketch of different medication modes. Prophylactic administration: drugs added to the cell culture before the infectious virus was added to A549 cells. Premixed administration: drugs and virus were mixed, incubated at 4°C for 1 h, and then added to the cell culture mixture. Simultaneous administration: Drugs and virus were mixed and immediately added to the cell culture. Therapeutic administration: drugs were added after infection by the virus to A549 cells. Green: the period of cell culture; Yellow: in this time, the drug was in the medium; Red: the time of the virus infection; Magenta: drugs and the virus in the medium at the same time; Blue: there was cell culture without drugs and virus; Black: this was the period of detection.

Columns 2–10 of the 96-well plate received dilutions with different concentrations of Asprellcosides B and Oseltamivir. The first column was the initial concentration; and the concentrations subsequently decreased, with the tenth column as the blank control. Asprellcosides B was added to rows 2–4 of the 96-well plate, and Oseltamivir was added to rows 5–8. Each concentration of drugs was repeated three times and then incubated for 48 h at 37°C, 5% v/v CO_2_ in a humidified incubator. The method of adding the drugs are shown in Figure 2.

#### WST-8 Assay

The viability of A549 cells was quantified by the Total Superoxide Dismutase Assay Kit with WST-8 (WST-8 assay) (Tahara et al., 2017) using a Cell Counting Kit-8 (Dojindo). Cells were incubated in 10% of WST-8 reagent in a volume of 100 μL at 37°C and 5% v/v CO_2_ for 30min. The absorbance was measured at 450 nm by a multimode microplate reader (PerkinElmer).

The cytotoxicity of Asprellcosides B was assessed by the WST-8 assay using a Cell Counting Kit-8 (Dojindo). A549 cells were grown in 96-well plates at 37°C and 5% v/v CO_2_ for 24 h and then incubated with different concentrations of Asprellcosides B for 48 h. The absorbance was then measured at 450 nm using a Multimode Microplate Reader.

#### Neuraminidase Assay

The inhibitory effects of Asprellcosides B on the influenza virus neuraminidase activity, were measured by a fluorescence assay (Sidwell and Smee, 2000). The reaction mixture, containing 50 μL of NA-Fluor^TM^ Substrate, 50 μL of the supernatant of cell and virus cultures, was incubated at 37°C for 1 h in 96-well microtiter plates. The reaction was terminated by adding 100 μL of NA-Fluor^TM^ Stop Solution to each well. The absorbance was then measured at 365/450 nm by the multimode microplate reader showing the content of neuraminidase.

#### RT-PCR Assay

The MDCK and Vero cells were grown to approximately 90% confluence, infected with 50 TCID_50_ of the influenza A and Zika virus, DEVN. The drugs were then continuously added. At 48 h post-infection, cells were collected and centrifuged (12,000 *g* for 5 min). The extraction of the total RNA from cells was carried out using the Ultrapure RNA kit (CoWin Biotech, Beijing, China), and cDNA was then synthesized from the total RNA using the M-MLV Reverse Transcriptase kit (Promega, Madison, WI, United States) with random primers and oligo (dT) primers. The PCR reactions system was performed with 25 μL reaction buffer (17 μL of RNA template, 5 μL of M-MLV 5× reaction buffer, 50 pmol of the primers, 0.1 mM dNTPs, 25units of ribonuclease inhibitor, and 200 units of M-MLV RT polymerase). The amplification conditions of RT-PCR analysis were 1 cycle at 95°C for 3 min; 39 cycles at 95°C for 10 s, 60°C for 10 s, and 72°C for 20 s; and 1 cycle at 95°C for 10 s.

The specific primers for HA mRNA and NP mRNA of A/FM/1/47(H1N1) to detect viral RNA were: 5′-CCCAGGAGATTTCGCCGACTATG-3′ and 5′-TGCCGTTACTCCTCTGGTTATGTTG-3′; 5′-CATCTTTCTGGCACGGTCTG-3′ and 5′-GGCTACTGCAGGTCCATACA-3′, while for GAPDH, which was used as an internal control, the primer sequences (Nogales et al., 2014) were 5′-AACATCATCCCTGCTTCCAC-3′ and 5′-GACCACCTGGTCCTCAGTGT-3′. The primers of the dengue virus were: 5′-CATTCCAAGTGAGAATCTCTTTGTCA-3′ and 5′-CAGATCTCTGATGAATAACCAACG-3′. The primers for the Zika virus were: 5′-GGTCAGCGTCCTCTCTAATAAACG-3′ and 5′-GCACCCTAGTGTCCACTTTTTCC-3′.

#### Plaque Reduction Assay

The MDCK cells were grown to approximately 90% confluence12-well plates, infected with supernatants for 2 h at 37°C. The supernatants collected from the cells were infected with 50 TCID_50_ of the influenza A/PuertoRico/8/34(H1N1) virus, then added to different concentrations of Asprellcosides B and incubated for 48 h at 37°C. The infected substance was then removed and cell was washed with PBS. The cell monolayers were overlaid with agar containing supplemented MEM (1% low melting point agarose, 1% bovine serum albumin and 1 μg/ml TPCK-treated trypsin) and incubated at 37°C for 2–3 days. The sample was fixed with 4% paraformaldehyde for 2 h and removed with an agarose overlay. Cells were then stained with 2% (w/v) crystal violet.

### Pharmacodynamic Mechanism Research

#### Time of Addition Experiments

The cells infected with the influenza virus were medicated at different time-points. After virus infection (50TCID_50_), cells were washed twice with 100 μL of (Phosphate Buffer Solution, PBS) (pH7.4) and added to a twofold gradient of Asprellcosides B, diluted with the infection medium containing 1% DMSO. The initial concentration was 300 μM and a total of four gradients were used. Dilutions of 100 μL were added at different time-points (-2–-1 h, -1–0 h, 0–2 h, 2–4 h, 4–6 h, and 4–48 h), and the negative control was 100 μL of the infection medium, containing 1% DMSO. Cells were then incubated for 48 h at 37°C, 5% v/v CO_2_ in a humidified incubator.

#### Hemagglutinin Inhibiting Assay

Samples were diluted twofold in a phosphate-buffered saline (PBS, pH7.4), in a V shape, in 96-well-plates. The samples had different concentrations of drugs, and the initial concentration was 500 μM. A total of 25 μL of the specimen and then 25 μL of the influenza virus was added to each sample (Inouye and Kono, 1972). After 30 min of incubation at 4°C, 50 μL of 1% suspension of chick erythrocytes was added to each well. The virus titer was TCID_50_ = 10^-2.5^/0.1 mL. Plates were incubated for 20 min at 37°C, and the agglutination could be observed with the naked eye.

#### Hemolysis Inhibiting Assay

The compound was diluted in PBS with the final concentration of 100 μM/mL, and 100 μL of the specimen dilution was added to the long well 96-well-plates. The blank control was a PBS solution. 100 μL of the undiluted virus stock was then added to the sample wells and the negative control wells, and the virus titer was TCID_50_ = 10^-2.5^/0.1 mL. The wells with samples were completely mixed, and the entire plate was incubated for 30 min at room temperature. 200 μL 2% suspension of chick erythrocytes was then added, which was preheated in a water bath at 37°C. The plates were then shaken for 60 s and were incubated at 37°C for 30 min. After the incubation, 100 μL of prepared solutions of acetic acid-sodium acetate at different pH values (4.6, 4.8, 5.0, 5.2, 5.4, 5.6, 5.8, and 6.0) were added and sufficiently mixed. Plates were then incubated in an incubator at 37°C for 30 min. After centrifugation at 1200 rpm for 6 min, the absorbance of the supernatant at 540 nm (Optical density at 540 nm, OD_540_) was detected.

#### Docking Simulation Analyses

The primary 3D structure of Asprellcosides B was drawn using Chemoffice 2015 software. Its conformation was optimized by the RDkit (Ebejer et al., 2012), and several low-energy conformers were selected for molecular dynamics simulation (MD) by AutoDockTools (Morris et al., 2009). The crystal structure of HA of A/WSN/33 H1N1 strains was analogous to the structure of A/Puerto Rico/8/1934 H1N1, and it was downloaded from the Protein Data Bank (PDB ID:1RU7 and 1RVX) (Gamblin et al., 2004).

Coordinate files were prepared using AutoDockTools. Docking of Asprellcosides B was performed using the HA1 sialic acid binding pocket by AutoDock 4.26. The docking areas were within 3 Å from the crystallization of the ligand and the 90-Helex, 130-Loop, 220-Loop sites (PD ID: 1RVX). Prediction of the binding site of Asprellcosides B and HA2 by Autoligand was performed (Russell et al., 2008; Song et al., 2016), and the terminal binding site was confirmed by molecular docking (PDB ID: 1RU7, 3EYM). Docking results were analyzed with AutoDockTools.

#### SPR Analyses

Kinetic information of the binding of Asprellcosides B and HA protein was obtained using a Biacore T100 instrument (GE Healthcare, Uppsala, Sweden). Hemagglutinin (HA) protein (Sino Biological INC., Beijing, China), influenza (A/WSN/33 H1N1 with His Tag) was immobilized on a CM7 sensor chip (GE Healthcare, Uppsala, Sweden) in 10 mM sodium acetate at pH4.5, using the amine coupling method. The chip density was 20,000 (Resonance Units, RU). The reference flow cell was used as a control for non-specific binding. Compounds of Asprellcosides B were gradually diluted in a Phosphate buffer solution and Polyoxyethylenesorbitan 20 (PBS-P: 10 mM phosphate buffers with 2.7 mM Potassium Chloride, KCl and 137 mM Sodium Chloride, NaCl, 0.05% surfactant P20, pH7.4) and 1% DMSO. The specimens were prepared by twofold dilution, with their concentrations ranging from 312.5 nM to 20 μM. Samples were injected for 180 s and the flow rate was 30 μL/min. The dissociation followed for 240 s and surface regeneration was with 50 mM NaOH for 30 s. Data were fit using a 1:1 binding model by Biacore T100 Evaluation software Version 2.0.3.

## Results

### Asprellcosides B Is an Ursane Type Triterpenoid Saponin With 3-*O*-Xyl, 28-*O*-β-D-Glucopyranoside

Asprellcosides B was a colorless tabular crystal (MeOH). Its molecular formula was C_41_H_66_O_16_S as a basis for (Electrospray Ionization Mass Spectrometry, ESI-MS) (m/z845 [M-H]^-^), ^13^C (Nuclear Magnetic Resonance, NMR) (100 MHz, Pyridine-d5), and ^1^H NMR (400 MHz, Pyridine-d5). The Infrared Radiation (IR) Potassium Bromide Tablet (KBr) spectral absorption peak of the compound was [3421 cm^-1^ (hydroxyl)], [2941, 2875, 1457, and 1387 cm^-1^ (methyl)], [1735 and 1646 cm^-1^ (ester group)], [1250 and 1072 cm^-1^ (sulfonic)], [876 and 586 cm^-1^ (Double bond hydrogen)], and the Under Voltage (UV) (MeOH) spectral absorption peak was approximately 206 nm; according to the elemental analysis, the sulfur content was 3.655%.

**FIGURE 3:**
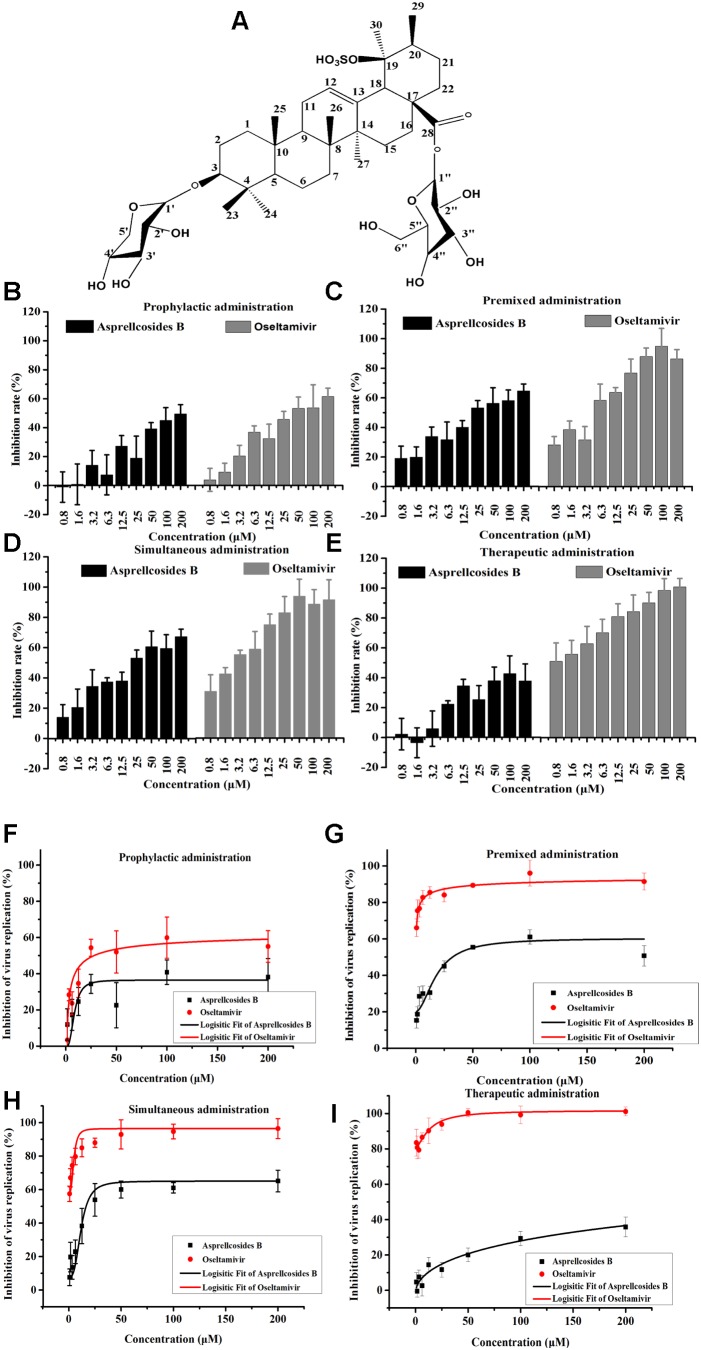
Asprellcosides B inhibited influenza A virus infection in A549 cells. **(A)** The structure of Asprellcosides B. **(B–E)** Different modes of medication. The cell survival rates of Asprellcosides B and Oseltamivir on influenza A/FM/1/47(H1N1) were determined by WST-8 assay at 48 h post-infection (*n* = 3, each concentration conducted in triplicate). **(F–I)** Different modes of medication. The inhibitory effects of Asprellcosides B and Oseltamivir on the neuraminidase activity of influenza A/FM/1/47(H1N1) were determined by fluorescence assay at 48 h post-infection. *n* = 3, each concentration conducted in triplicate.

The ^1^H NMR and ^13^C NMR spectra showed that there were seven methyl groups [δ_H_ 1.41, 0.98, 0.88, 1.18, 1.73, 1.29, and 1.0 (3H, S, 7 × CH_3_); δ_C_ 28.1 (C-23), 16.5(C-24), 15.3(C-25), 17.1(C-26), 15.8(C-27), 29.4(C-29), and 24.0(C-30)], seven quaternary carbons [δ_C_ 39.1(C-4), 40.1(C-8), 36.6(C-10), 138.5(C-13), 42.5(C-14), 48.0(C-17), and 41.78(C-19)], one ester carbonyl (δ_C_ 176.7), and two alkenyl groups [δ_C_ 127.25(C-12) and 138.5(C-13)]. The analysis of [M-H]^-^ ion in the negative ion HRESIMS (m/z 845, m/z 683), ^1^H NMR and ^13^C NMR spectra displayed that they were 3-*O*-xyl [δ_H_ 4.60, d (7.2), 6.35, d (7.6), (α:4.77, β:3.76,(dd)); δ_C_ 106.6(C-1′), 73.3(C-2′), 73.7(C-3′), 67.8(C-4′), 66.4(C-5′)], and 28-*O*-β-D-glucopyranoside [δ_C_ 95.49(C-1″), 73.0(C-2″), 78.7(C-3″), 70.7(C-4″), 78.9(C-5″), 61.8 (C-6″); δ_H_ 6.35, d (7.6), 4.24, t (8.5), 4.32, t (8.5), 4.45 (m), 4.21 (dd), (α:0.79, β:1.43)]. Thus, the structure of the compound was established as an Ursane type triterpenoid saponin with 3*-O*-xyl, 28-*O*-β-D-glucopyranoside (Zhou et al., 2008, 2012; Yao et al., 2009) (Figure 3A).

### Asprellcosides B Represses Influenza A Virus Infection in A549 Cells

The cytotoxicity and antiviral activity of Asprellcosides B in the regulation of the influenza virus H1N1 A/FM/1/47(H1N1) in A549 cells, were determined by Neuraminidase (NA) inhibition assays and WST-8 cell proliferation and cytotoxicity assays. There was a low toxicity of Asprellcosides B in confluent A549 cells, monolayers with concentrations of 1.03–250 μM, and the TC_50_ was >250 μM. With different modes of medication, Asprellcosides B displayed anti-influenza virus activity (Table 1 and Figure 3). The cytopathic effect (CPE) was observed at 48 h post-infection. At the same time the determination of cell viability (CPE inhibition) was employed to represent the inhibition rate by using the WST-8 assay (Figures 3B–E). According to the data, the inhibitory effect of Asprellcosides B in Premixed administration and Simultaneous administration were superior in terms of dose-dependence. The inhibitory effect of 25 μM Asprellcosides B was over 50%.

**Table 1:** *In vitro* anti-viral activity against H1N1 A/FM/1/47 by Asprellcosides B and *Ilex asprella* (Hook. Et Arn.) Champ. *Ex Benth* isolated from the radix and stems, with different medication modes.

Virus	Therapy	Asprellcosides B		Oseltamivir	
		TC50 > 250 μM		TC50 > 250 μM	
			
		IC50 (μM)	SI (μM)	IC50 (μM)	SI (μM)
H1N1	Therapeutic administration	796.46	0.31	13.21	18.92
	Simultaneous administration	11.21	22.30	4.26 × 10^-5^	5.87 × 10^6^
	Premixed administration	18.39	13.59	1.64 × 10^-5^	1.52 × 10^7^
	Prophylactic administration	8.87	28.18	6.10	40.98


*The TC50 and IC50 values were determined by WST-8 assay and neuraminidase assay. n = 3, each concentration in triplicate*.

With an intention to identify the inhibitory effect of Asprellcosides B on influenza virus infection, Asprellcosides B mediated inhibition of viral protein synthesis in A549 cells was examined through Neuraminidase assay. We analyzed virus neuraminidase activity using NA-Fluor Substrate at 48 h post-infection. In Premixed administration and Simultaneous administration, the inhibitory effect of Asprellcosides B displayed higher dose-dependence (Figures 3D,E). In Prophylactic therapy, Neuraminidase assay featured Asprellcosides B inhibited cell viability with IC50 above 8.87 μM (Figures 3F–I and Table 1); hence, the selection index (SI) exceeded 28.18, indicating that Asprellcosides B may possess a promising safety profile. The solubility of Asprellcosides B in water was only 1.66 μg/mL. Consequently, it did not reach a complete inhibition even at 200 μM as Oseltamivir did. This result might be attributed to its low solubility.

### Asprellcosides B Attenuates Influenza A Virus mRNA Transcription in MDCK Cells

**FIGURE 4:**
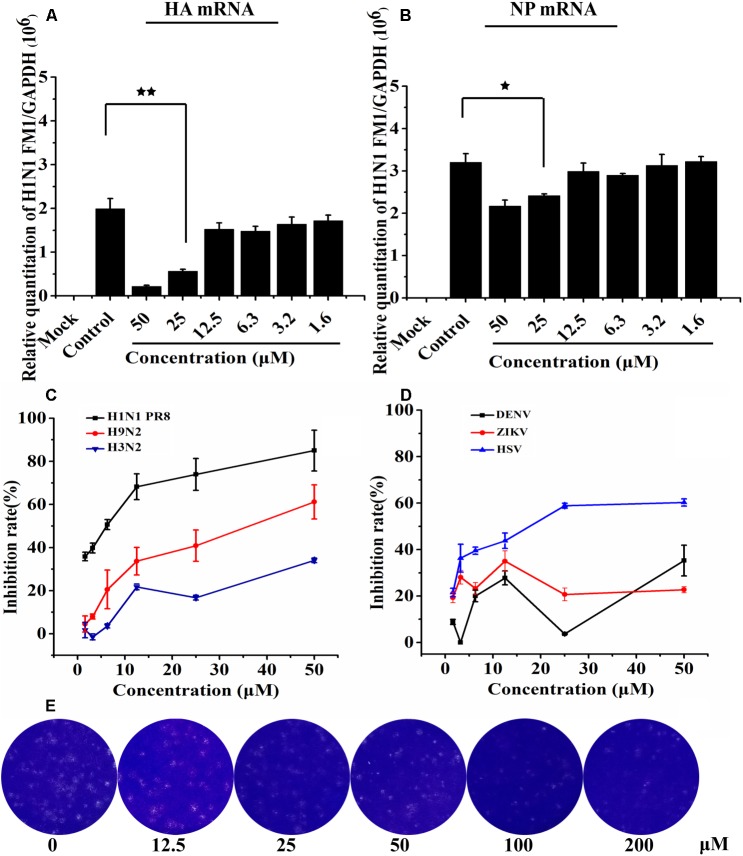
Asprellcosides B attenuates influenza A virus mRNA transcription in MDCK cells. **(A,B)** The inhibitory effect of Asprellcosides B on the expression of influenza A/FM/1/47(H1N1) HA mRNA and NA mRNA were detected by quantitative real-time PCR at 48 h post-infection. ^∗∗^*p* < 0.01, ^∗^*p* < 0.05. Assessed the antiviral activity of other influenza virus. **(C)** The antiviral activity of H1N1 PR8, H9N2, H3N2, and HSV virus were assessed through CPE inhibition by using WST-8 assay, and **(D)** the antiviral activity of ZIKA and DEVN virus was determined by quantitative real-time PCR. **(E)** The plaque was fixed and stained at 48 h post infection, and the experimental method was detailed in the Section “Materials and Methods.”

We conducted a quantitative real-time PCR to determine how the synthesis of viral mRNA in MDCK cells was inhibited by Asprellcosides B. Isolated from the infected cells, the total RNA was investigated by quantitative real-time PCR using primers of the viral HA mRNA and NP mRNA at 48 h post-infection. Upon the results, HA mRNA and NP mRNA transcription declined as a result of adding Asprellcosides B to influenza-virus-infected cells in a dose-dependent manner (Figures 4A,B). We found that Asprellcosides B had a significant effect in the inhibiting infection by A/PuertoRico/8/34 (H1N1), A/Chicken/Guangdong/1996 (H9N2) and, HSV virus, but less inhibition on the A/HongKong/498/97(H3N2) (Figure 4C), respectively, while it had no inhibition on the infection by ursane type triterpenoid the Dengue virus (DENV) and Zika virus (ZIKV) (Figure 4D). The results demonstrate that Asprellcosides B specifically represses the mRNA transcription of influenza A virus. The plaque reduction assay showed that Asprellcosides B can reduce the plaque numbers in a dose-dependent manner (Figure 4E).

### Asprellcosides B Represses Influenza A Virus Replication at the Early Stage of Its Lifecycle

**FIGURE 5:**
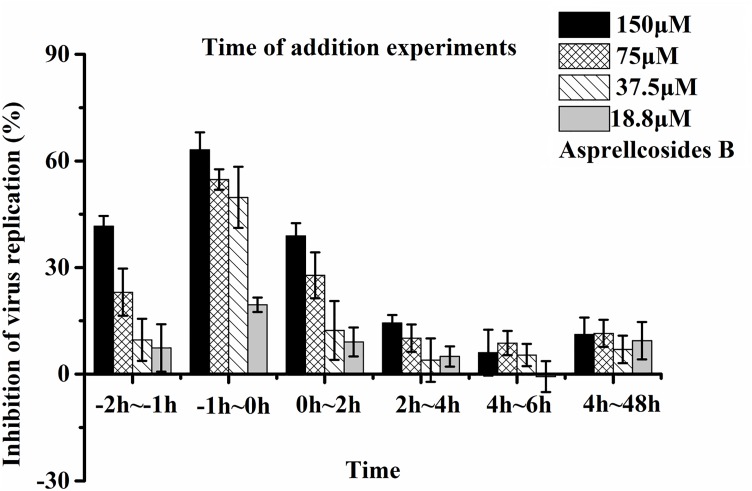
Asprellcosides B represses influenza A virus replication at the early stage of its lifecycle. Cells were inoculated with 50TCID_50_: 0.1 mL of the influenza virus A/FM/1/47(H1N1) in a time-of-addition assay. 18.8–150 μM of Asprellcosides B was added at the designated times and was removed after each incubation period. The cells were then incubated with fresh media until 48 h post infection. The virus replication was determined through Neuraminidase assay. *n* = 3, each concentration performed in triplicate.

Time-of-addition and time course studies concerning the inhibitory effects of Asprellcosides B were carried out to recognize the steps of Asprellcosides B inhibiting the influenza lifecycle. It has been reported that at least 6 h are required to detect a progeny virus after inoculation of the influenza A/PR/8/34 virus (Li et al., 2009). There were six intervals (-2–-1 h, -1–0 h, 0–2 h, 2–4 h, 4–6 h, and 4–48 h) selected for the inhibition time course, which determined the expression of the NA protein. The addition of Asprellcosides B to the cells within -1–0 h of viral infection in A549 cells presented an evident inhibition of virus replication (Figure 5). Asprellcosides B failed to demonstrate its antiviral activity when it was added more than 2 h after the infection (Figure 5), implying that Asprellcosides B is effective during the early stages, viral binding and the fusion and entry of the viral lifecycle. In summary, the above-mentioned results provides evidence that Asprellcosides B exerts an inhibitory effect on the early stages of the influenza virus lifecycle. It is worth mentioning that the inhibitory effect of Asprellcosides B on viral replication may be performed by targeting the hemagglutinin protein, because the Premixed administration and Simultaneous administration of the A549 cells with Asprellcosides B markedly impair the infection of the influenza virus.

### Asprellcosides B Inhibits Influenza A Virus HA-Mediated Hemolysis

**FIGURE 6:**
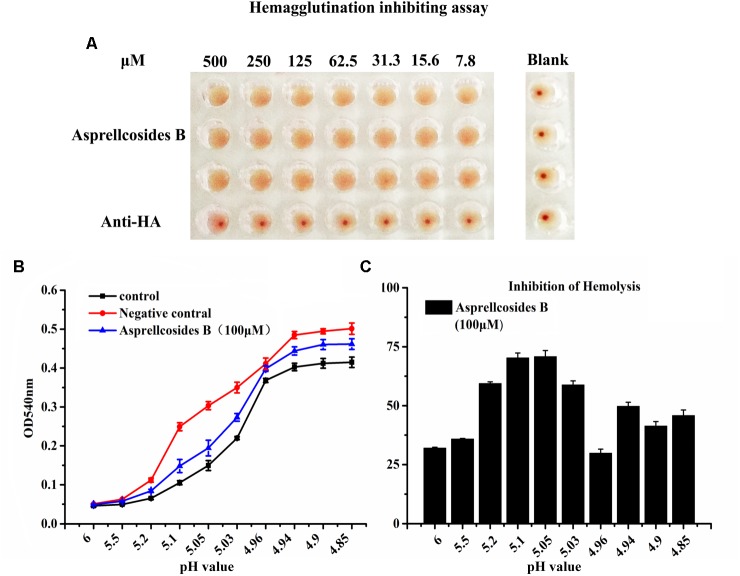
Asprellcosides B inhibits influenza A virus HA-mediated hemolysis. **(A)** 25 μL influenza A/FM/1/47(H1N1) virus was incubated at room temperature with a twofold gradient of diluted Asprellcosides B for 30 min. Added with dilutions of 100 μL at different time-points, the virus was subsequently incubated at room temperature for 48 h. The blank control was a PBS solution. Note the inhibition on hemagglutinin of Asprellcosides B. **(B)** Hemolysis inhibition assay of Asprellcosides B against the influenza A/FM/1/47(H1N1) virus strain. Asprellcosides B was diluted by PBS and was mixed in a 96-well-plate with an equal volume of the influenza virus A/FM/1/47(H1N1). 1% suspension of chicken erythrocytes was added after incubation at room temperature for 30 min. The plates were incubated for 20 min at 37°C. Then, sodium acetate at different pH values (4.6, 4.8, 5.0, 5.2, 5.4, 5.6, 5.8, and 6.0) was added and incubated at 37°C for 30 min to bring about hemolysis. At the end of incubation, the plates were centrifuged at 12,000 rpm for 6 min, and the supernatants were detected. A microtiter plate reader was used to read the OD_540__nm_. *n* = 3, each pH value in triplicate. **(C)** The inhibition ratios (%) of hemolysis of influenza virus H1N1 A/FM/1/47 with different pH values by Asprellcosides B.

For the purpose of disclosing the exact domain targeted by Asprellcosides B, a hemagglutinin inhibition assay was devised to observe whether the interaction between HA and cellular receptors could be interfered by ursane type triterpenoid of Asprellcosides B, after which virus attachment to the cell could be blocked (Figure 6A). The data showed that Asprellcosides B did not inhibit viral attachment to the cell, which was primarily intervened by the HA subunit. Moreover, whether viral-cell membrane fusion, which was mediated by HA2 subunit, could be interfered with by Asprellcosides B still requires further study.

Therefore, we designed the hemolysis assay with the influenza virus H1N1 A/FM/1/47(H1N1) to identify the effect of Asprellcosides B on fusion. To trigger hemolysis, the virus-cell suspension was briefly acidified to different pH (pH4.85–6.0) to initiate HA conformational changes that lyse chicken red blood cells (cRBCs). Wells absent in the virus were used as controls to determine the effect of compounds on cRBCs. The results revealed that in the virus control group, OD value increased rapidly when pH < 5.2, while OD value remained steady when pH < 4.94; when pH < 5.2, OD value in the blank control group and Asprellcosides B group raised progressively as PH declined; when pH < 4.94, OD value stayed stable, but its growth level in the blank control group and Asprellcosides B group was lower than that in the virus control group, while the Asprellcosides B group was similar to that of the blank control group (Figures 6B,C). While experimenting with the inhibition ratio of hemolysis by Asprellcosides, it was found that the virus content reached its highest level at a pH of 5.05, and the drug’s inhibition ratio arrived at 70.83%. Considering the results, Asprellcosides B could have an inhibitory influence on influenza-virus-HA mediated hemolysis, which suggests that the membrane fusion process was targeted by Asprellcosides B through virus entry.

### Asprellcosides B Directly Interacts With Hemagglutinin

**FIGURE 7:**
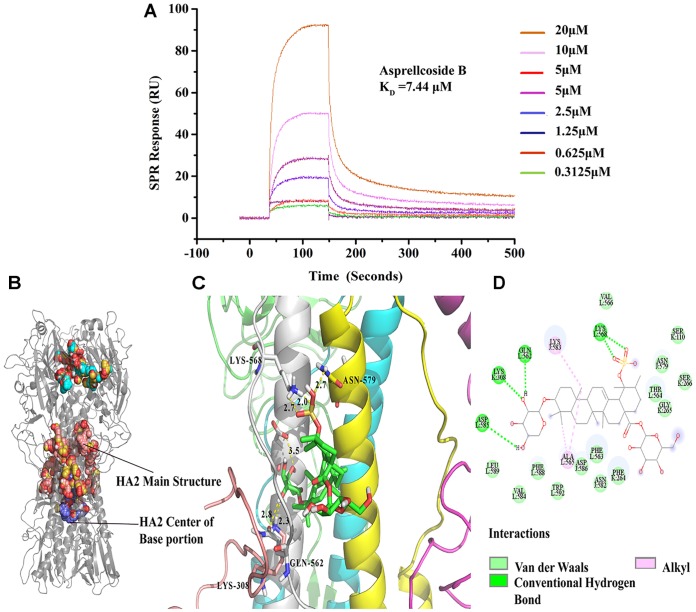
Asprellcosides B directly interacts with hemagglutinin. **(A)** The binding curve of Asprellcosides B with HA protein in SPR assay. **(B,C)** The 3D drawing of the estimated domain of the HA2 protein bound to Asprellcosides B. **(D)** The 2D drawing of the calculated domain of the insertion of Asprellcosides B in the HA2 protein-binding site.

Molecular docking simulation displayed the highest probability of the binding between Asprellcosides B and HA. Asprellcosides B was bound to the HA2 main structure with multiple non-polar bonds, and the glycoside and sulfate groups of Asprellcosides B were hydrogen-bound to lysine (L:568), glutamine (L:562), lysine (K:308), and aspartic acid (L:585). The L peptide chain and K peptide chain were split from the same HA2 protein (Figures 7B–D). The binding sites were similar to tert-butyl hydroquinone (TBHQ) (Yao et al., 2009), which is an inhibitor that binds to the hydrophobic pocket of HA.

It has been hypothesized that a binding affinity between HA protein and Asprellcosides B is present, and we confirmed it by a surface plasmon resonance (SPR) assay designed to determine the binding between two molecules. SPR analyses showed that there was a good kinetic affinity between Asprellcosides B and the HA protein, and the resonance units of the drug were present at concentration dependence. A 1:1 binding model was used, and the dissociation constant (KD) was calculated from the curve (Figure 7A). The association rate constant *K*_a_ was 583.5(1/Ms), and the dissociation rate constant *K*_d_ was 0.004343(1/s). Then, the affinity constant KD value (ratio *K*_d_/*K*_a_) was calculated to be 7.44 μM.

## Discussion and Conclusion

Influenza A virus infection is a major contributor to yearly mortality worldwide. It poses a grave concern for loss of life in influenza pandemics, exemplified by the 1918 Spanish flu that killed around 50 million people. Current antiviral therapies using NA inhibitors are inadequate to control the spread of the influenza virus caused by the rapid emergence of drug-resistant mutations. There is an urgent need to discover and develop more efficacious antiviral drugs that target various viral proteins essential for infection and replication. The HA is essential for mediating virus attachment to cells and inducing membrane fusion between viral and cellular membranes and therefore is an ideal antiviral target.

In this study, we have demonstrated that Asprellcosides B greatly inhibited influenza A virus infection in cell cultures with an EC50 of about 9 μM. It was not toxic to cells up to 250 μM with a selective index of greater than 28. The time-of-addition experiments suggested that Asprellcosides B acted on an early step of virus infection, possibly at the membrane fusion step (Ackermann and Maassab, 1954). The HA is essential for virus attachment and membrane fusion between viral and cellular membranes upon the low pH-induced conformational change (Du et al., 2013). Several previous studies have identified inhibitors of the receptor binding or membrane fusion activities of HA (Deshpande et al., 2001; Yao et al., 2009). Our results also suggest that the HA is the molecular target of Asprellcosides B. Asprellcosides B did not affect hemagglutination *in vitro* but significantly inhibited the virus-mediated and low pH-induced hemolysis. This conclusion is also supported by the molecular docking suggesting a multi-hydrogen-bound interaction between the glycoside and sulfate groups of Asprellcosides B and the HA2. Similarly, the interaction between Asprellcosides B and the HA2 was confirmed by a surface Plasmon resonance analysis, demonstrating high affinity binding. In summary, Asprellcosides B inhibited the influenza A virus infection in cell cultures, through a specific interaction with the HA2, resulting in a blockade of the HA-mediated membrane fusion.

## Author Contributions

GL conceived and designed the experiments. WZ, S-TC, Q-YH, L-QH, XL, S-FZ, H-TH, and X-HL analyzed the data. X-PL and JW contributed reagents, materials, and analysis tools. WZ wrote the manuscript. All authors participated in the work and read and approved the final manuscript.

## Conflict of Interest Statement

The authors declare that the research was conducted in the absence of any commercial or financial relationships that could be construed as a potential conflict of interest.
